# Thyasirid species composition (Bivalvia: Thyasiridae) and genetic connectivity of *Parathyasira equalis* (A. E. Verrill & K. J. Bush, 1898) in deep basins of sub-Arctic fjords

**DOI:** 10.1186/s12862-024-02278-3

**Published:** 2024-07-04

**Authors:** Valentin Kokarev, Suzanne C. Dufour, Joost A. M. Raeymaekers, Amalia A. Mailli, Henning Reiss

**Affiliations:** 1 Department of Biology, Memorial University of Newfoundland, St. John’s, Newfoundland, Canada; 2 Faculty of Biosciences and Aquaculture, Nord University, Bodø, 8049 Norway

**Keywords:** Norwegian coast, Macrobenthic communities, Infauna, Dispersal, Genotyping-by-sequencing (GBS), Genetic structure, Metacommunity

## Abstract

**Background:**

Thyasirid bivalves are often recorded as a dominant component of macrobenthic infaunal communities in depositional environments such as fjord basins. Fjord basins comprise patchy soft-bottom habitats bounded by steep walls and sills; however, little is known how this semi-isolated nature of fjords affects benthic populations. Accordingly, data on the composition and population connectivity of thyasirids can provide valuable information on the ecology of these ecosystems.

**Results:**

The species composition of thyasirid bivalves has been studied in the basins of three sub-Arctic fjords (Nordland, Northern Norway). Overall, six thyasirid species were recorded: *Parathyasira equalis*, *Parathyasira dunbari*, *Mendicula ferruginosa*, *Genaxinus eumyarius*, *Thyasira sarsii*, and *Thyasira obsoleta*. The species composition remained stable within the basins during the sampling period (2013–2020) and suggested the importance of local reproduction over advection of individuals for population dynamics. Only one species, *Parathyasira equalis*, was common in all fjords. We have further investigated the population genetics of this species by combining two types of genetic markers: a 579 bp fragment of the cytochrome c oxidase subunit I (COI) gene and 4043 single-nucleotide polymorphisms (SNPs) generated by genotyping-by-sequencing. The latter provided a more in-depth resolution on the population genetics of this species and revealed a weak but significant differentiation of populations within fjords, further indicating limited connectivity between basins.

**Conclusion:**

Based on our findings, we conclude that limited dispersal between the basin communities results in weakly connected populations and might be an important structuring factor for macrobenthic communities.

**Supplementary Information:**

The online version contains supplementary material available at 10.1186/s12862-024-02278-3.

## Background

Bivalves of the family Thyasiridae are infaunal organisms that burrow within sediments with a vermiform foot and are often associated with reducing habitats such as areas with high input of organic matter and methane seeps [1–3]. As an adaptation to these habitats, many larger thyasirid species are known to form a symbiotic relationship with chemosynthetic sulfur-oxidizing bacteria, while smaller species (shell length of 2–3 millimeters) generally derive their nutrition heterotrophically [4, 5]. Populations of symbiotic thyasirid species that rely on the sulfur-oxidizing bacteria are often dependent on relatively high concentrations of hydrogen sulfide in the sediment, but other species are sensitive to increased sulfide concentrations [6]. These autecological differences between symbiotic and asymbiotic species are important drivers of their spatial distribution [7]. In addition to autecological preferences, local spatial distribution and population dynamics of thyasirids might be driven by dispersal. Thyasirids are a dominant component of infaunal communities in fjord basins, where basin topography results in the patchiness of soft-bottom habitats [8, 9]. In such spatially structured habitats, dispersal can play an important role both in determining local population dynamics as well as processes at the community level [10–12]. The restricted exchange with adjacent waters due to the presence of a sill at the mouth might act as a dispersal barrier, limiting the dispersal of individuals among populations (connectivity) and potentially affecting population dynamics and species composition of thyasirids.

For most benthic sessile and sedentary species, population connectivity is thought to be achieved through a pelagic larval stage [13]. However, many benthic species have demersal lecithotrophic larvae or direct development, and consequently, limited dispersal capabilities as opposed to planktotrophic larvae that can spend up to several months in the water column [14]. In bivalves, the size of the larval shell, or prodissoconch, is often used as an indication of development type when biological data and direct observations are not available [15]. Currently, biological data on the development of thyasirids are rather scarce: for *Thyasira gouldii*, with a prodissoconch size of > 200 μm, direct development with no pelagic stage has been documented, while for the species with smaller prodissoconch sizes (< 190 μm), such as *Thyasira flexuosa*, a short pelagic life stage was suggested, although its duration is not known [16]. Thus, it can be assumed that thyasirid bivalves, similarly to other benthic infaunal species with lecithotrophic development, mainly disperse with near-bottom currents [17]. Consequently, water exchange below the sill depth might be an important factor determining the dispersal and population connectivity of thyasirid bivalves in the deep basins of fjord systems.

The connectivity of populations can be studied with a variety of approaches such as biophysical modelling of larval dispersal or genetic markers [13, 18]. Usually, limited connectivity among populations in spatially restricted habitats leads to population genetic differentiation due to reduced gene flow [19]. Recently, along with traditionally used markers such as fragments of mitochondrial genes and microsatellites, genome-wide approaches such as restriction site associated DNA (RAD) and genotyping-by-sequencing (GBS) considerably increased the resolution of population studies by increasing the number of genetic markers, providing data on more than 1000 single-nucleotide polymorphisms (SNPs) in non-model organisms [20]. Genome-wide approaches can complement mitochondrial DNA data while also providing new insights into population connectivity patterns among marine invertebrates, even at fine scales [21–25]. However, a relatively poor understanding remains of how gene flow in fjords is affected by the combination of circulation patterns, associated with basin geomorphology and sills, along with species biology and migration patterns. Several studies based on fish suggest that fjord fish populations are isolated [26–28]. Some species of zooplanktonic copepods have genetically distinct resident populations in fjords, while drifting species show less pronounced genetic structure [29–31]. Similarly, no apparent population structure was evident for the deep-water jellyfish *Periphylla periphylla* along the Norwegian coast [32]. However, previous studies in the fjords were mostly based on microsatellite and mitochondrial markers and rarely assessed the population connectivity of benthic species. The marker type might be particularly important for understanding the patterns of genetic connectivity [33]. Population studies utilizing more advanced methods, such as GBS, will be crucial for improving the understanding of population connectivity in fjords as well as providing additional baseline information for ecosystem management [34].

In the present study, we studied thyasirid bivalve populations in the three fjord basins in Northern Norway. Previous work on macrobenthic communities in the area found a significant differentiation of macrobenthic communities in the basins while the fjord with a shallow sill was the most distinct in terms of community composition [8]. However, the dispersal pattern between the basins remains unclear, as well as its potential role in structuring macrobenthic communities relative to environmental drivers. We aimed to investigate the potential role of dispersal by addressing processes on the population level of thyasirid bivalves, a dominant taxonomic group in the infaunal communities. First, we describe the thyasirid species composition over the sampling period (2013–2020) considering autecological knowledge of different species. We hypothesize that limited dispersal between basins would lead to temporal stability of species composition as local reproduction would be more important than the advection of individuals from nearby habitats. Secondly, we assess the population connectivity between the basins by addressing the genetic differentiation of the most common thyasirid bivalve in these fjords, *Parathysira equalis*.

## Materials and methods

### Study area and study design

**Fig. 1:**
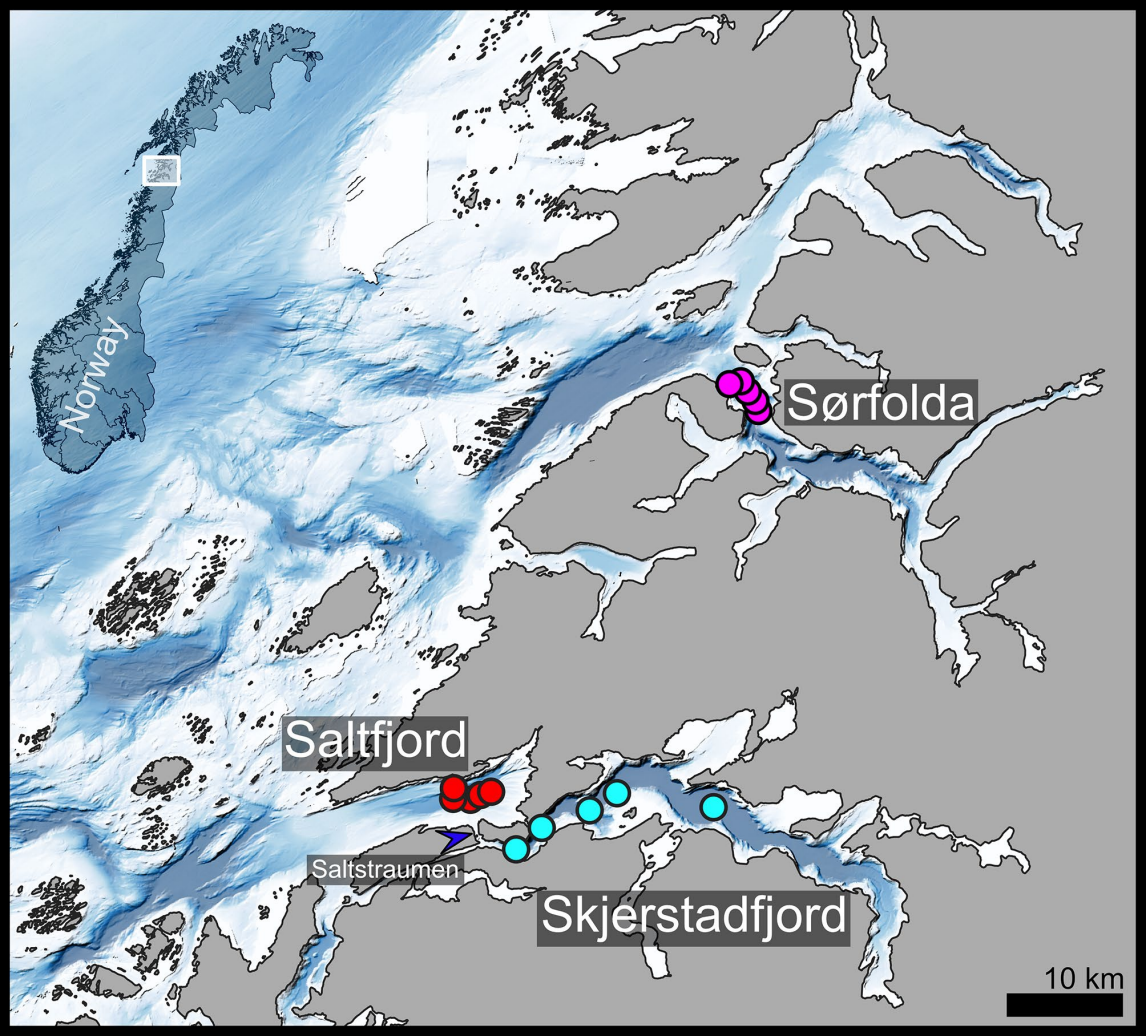
Map of the study area. Circles represent the sampling locations of thyasirids in the Fall of 2020. The location of a shallow sill, Saltstaumen, is indicated by a blue arrow. The coordinates for each sampling station are available in Supplementary Material 1

We have sampled three sub-Arctic fjords located at 67 °N in Nordland, northern Norway (Fig. 1). Sørfolda is located approximately 40 km north of Saltfjord-Skjerstadfjord. Saltfjord and Sørfolda are separated by relatively deep sills (220–260 m) from the adjacent waters, which allows for the inflow of Atlantic waters into their basins [8]. Saltfjord is the shallowest of the three fjords studied (maximum depth 380 m). The main route of water exchange between Saltfjord and adjacent deeper Skjerstadfjord (maximum depth 540 m) is the shallow and narrow strait Saltstraumen (max. depth 26 m). Saltstraumen is a hydrologically active area through which significant amounts of water enter Skjerstadfjord, forced by tidal currents [8, 35]. This water exchange is limited to the upper water layers: on the rising tide, the surface waters from Saltfjord enter Skjerstadfjord and sink to the deeper layers due to the differences in densities, while Skjerstadfjord waters entering Saltfjord on the falling tide stay on the surface due to higher freshwater content. Consequently, warmer and more saline waters of Atlantic origin, that form the bottom water layer (200–380 m) in Saltfjord [36], do not enter Skjerstadfjord. As a result, the bottom water layer of Skjerstadfjord (4.9 °C and 33.8 psu in May 2015) is several degrees colder and less saline compared to the bottom water layer of Saltfjord (7.3 °C and 35.3 psu in May 2015). Sørfolda (maximum depth 560 m) has bottom water properties similar to Saltfjord (7.0 °C and 35.3 psu in May 2015). The silty sediments are relatively homogenous within the basins in terms of granulometric composition [8]. While there is no single definition or defining criterion for “population” [37, 38], we assumed that groups of the same species inhabiting the same basins can be considered as populations due to distinct spatial disjunctions between the habitats. We hypothesized that the pattern of water exchange between Saltfjord and Skjerstadfjord would hamper the population connectivity of sedentary thyasirid bivalves with demersal larval life stages. Therefore, the populations sampled in the Saltfjord-Skjerstadfjord system would reveal possible isolation by a shallow sill, while the population in Sørfolda would represent possible isolation by distance.

### Thyasirid sampling

Thyasirid bivalves for genetic analysis were sampled with R/V “Tentayen” during fall 2020 using a 0.1 m^2^ Van Veen grab and sieved on a 1 mm steel mesh (6 samples in Saltfjord, 5 samples in Sørfolda, 6 samples in Skjerstadfjord). Thyasirids were sorted out from the remaining sediment live, identified, and photographed prior to dissection. The coordinates for each sample as well as numbers of retrieved thyasirid bivalves are available in Supplementary Material 1. It should be noted that these samples were processed rapidly, and consequently some of the smaller bivalves may have been lost, making a direct comparison with quantitative grab samples impossible. Valves were separated with a razor blade and soft tissues were preserved at -80 °C (*Parathyasira equalis*) or in 100% ethanol at -20 °C (other thyasirids) for further extraction of DNA. In addition, we used data on the distribution of thyasirids from the quantitative grab samples collected from these fjords in 2013 and 2015 to assess the temporal stability of thyasirid populations [8 and unpublished data]. The quantitative 0.1 m^2^ grab samples were fixed with buffered 4% formalin solution in seawater. Animals from the samples were sorted from sediments, identified to the lowest taxon possible, counted, and stored in 70% ethanol. Only stations that corresponded to the stations sampled in 2020 were used, e.g. stations F1-F2 and S10-S12 from [8] were not used. The coordinates of the samples and thyasirid densities for the sampling of 2013 and 2015 are available in Supplementary Material 1.

### DNA extraction

DNA was extracted from frozen (-80 °C, *Parathyasira equalis*) or ethanol-preserved (other thyasirids) tissues using Qiagen QIAamp DNA Mini Kit or DNeasy Blood & Tissue kits following the manufacturer’s protocol. The concentration of isolated DNA was measured with a Qubit 3 fluorimeter (Thermofisher Scientific). Overall, 86 DNA extracts (76 for *Parathyasira equalis* and 10 for other thyasirids) were used in this study (Supplementary Material 2).

### Sanger sequencing of 28S and COI gene fragments

The extracted DNA was then used as a template for the amplification of a 667 bp fragment of the cytochrome c oxidase subunit I (COI) and a 700 bp fragment of the 28S rRNA nuclear gene by polymerase chain reaction (PCR) (Table 1). Both genes were used to confirm the species identity by comparison with available sequences in GenBank. For the amplification of the COI fragment, the primers BivF4-t1 and BivR1-t1 were used [39, 40]. For the amplification of the 28S fragment, the primers LSU900f and LSU1600r [41] were used. PCR reactions were prepared separately for each marker, following the same recipe in a total volume of 25 µL, including 12.5 µL of the AccuStart II PCR ToughMix (2X) (from Quantabio), 7 µL of ultrapure nuclease-free water, 0.5 µL of MgCl_2_, 0.5 µL of each primer (Forward and Reverse) concentrated at 10 µM, and 4 µL of extracted DNA. PCR was performed in a Veriti 96-Well Fast Thermal Cycler (Applied Biosystems), starting with an initial 3 min long denaturation at 94 °C, followed by 38 cycles including 1 min of denaturation at 94 °C, 1 min of annealing at 52 °C, 2 min of elongation at 72 °C, and one final elongation step of 7 min at 72 °C. After the PCR was completed, 5 µL of each amplicon were immediately cleaned with 2 µL of ExoSAP-IT TM PCR product cleanup reagent (Applied Biosystems) in a reaction performed in a thermocycler for 30 min at 37 °C and 15 min at 80 °C. To prepare cleaned amplicons for sequencing, a BigDye Terminator reaction was carried out on ice in a final volume of 10 µL with 1 µL of BigDye Terminator v3.1 (Applied Biosystems), 1 µL of sequencing buffer, 1 µL of primer (Forward or Reverse, at 5 µM), 4 µL of ultrapure water and 3 µL of ExoSAP-cleaned PCR products. Reactions were performed in both Forward and Reverse directions in a Veriti 96-Well Fast Thermal Cycler (Applied Biosystems), starting with a 5-minute-long denaturation at 96 °C, followed by 28 cycles including 10 s. at 96 °C, 5 s. at 50 °C and 4 min at 60 °C. Unincorporated dye terminators, as well as any remaining nucleotides, salt, and primers, were removed using Mag-Bind SeqDTR (Omega Bio-tek) magnetic beads following the manufacturer’s protocol. Final PCR products were then dried by speed-vacuuming at 60 °C and resuspended in 10 µL of formamide. Sequencing was carried out on a 3500xL Genetic Analyzer (Applied Biosystems). The resulting sequences were quality checked (low-quality sequences were discarded), trimmed, manually corrected for misreads, and forward/reverse sequences were merged into contigs in CodonCode Aligner 9.0.2. All the sequences for 28S and COI obtained in this study were uploaded to GenBank (Table 1).

**Table 1:** Overview of the 28S and COI gene fragments that were obtained in this study. The coordinates and depth for each individual sample are available in Supplementary Material 2. Note: no successful sequences of COI could be obtained for *Thyasira sarsii.* The COI sequence for *Parathyasira dunbari*, and 28S sequences for *Thyasira obsoleta*, *Genaxinus eumyarius*, and *Parathyasira dunbari* are new to GenBank.

	28S		COI	
	Number of individuals	GenBank accession numbers	Number of individuals	GenBank accession numbers
*Parathyasira equalis* (A. E. Verrill & K. J. Bush, 1898)	6	PP333929-PP333934	69	PP334022-PP334090
*Parathyasira dunbari* (Lubinsky, 1976)	3	PP333941-PP333943	1	PP334095
*Thyasira obsoleta* (A. E. Verrill & K. J. Bush, 1898)	2	PP333939, PP333940	2	PP334093, PP334094
*Thyasira sarsii* (R. A. Philippi, 1845)	2	PP333936, PP333944	n/a	n/a
*Genaxinus eumyarius* (M. Sars, 1870)	2	PP333937, PP333938	1	PP334092
*Mendicula ferruginosa* (Forbes, 1844)	1	PP333935	1	PP334091

### Population genetics of *Parathyasira equalis* based on COI sequences

COI sequences of *Parathyasira equalis* were used to study the population genetics of this species. We assigned individuals to populations *a priori* based on the sampled basin: Saltfjord (30 individuals), Skjerstadfjord (24 individuals), and Sørfolda (15 individuals). In addition, 16 sequences of *Parathyasira equalis* from the fjords of Western Norway (60 °N) were downloaded from GenBank [42] to be used as an outgroup. The obtained COI sequences of *Parathyasira equalis* were trimmed and aligned in MEGA11 using the MUSCLE algorithm with default parameters [43]. The alignment of a total of 85 sequences of the 579 bp COI fragment was imported into PopART to produce a TCS haplotype network [44, 45]. In addition, an analysis of molecular variance (AMOVA) test was carried out in PopART to test for differences between populations. The results of AMOVA are reported as *Phi*-statistics (ratio of variance explained by factor “population” relative to total variance) and its *p*-value based on 1000 permutations.

### GBS sequencing and data analyses

In addition to population studies of *Parathyasira equalis* based on COI sequences, we performed genotyping-by-sequencing (GBS) for *de novo* SNP detection in this species. Overall, 44 samples of *Parathyasira equalis* were selected (16 from Saltfjord, 16 from Skjerstadfjord, and 12 from Sørfolda) based on the DNA yield (> 400 ng).

GBS library preparation, sequencing, and SNP calling were carried out by LGC Genomics Ltd. (Berlin, Germany). The complete protocol is available in Supplementary Material 3. In short, genomic libraries were prepared using the restriction enzyme MsII and sequenced on the Illumina NextSeq 500/550 V2 platform (150 bp paired-end reads with ~ 1.5 million read pairs per sample). The raw fastq files are available in Sequence Read Archive (BioProject: PRJNA1091663). Restriction enzyme combined reads were clustered with CD-HIT-EST v4.6.1 [46, 47]. Quality trimmed reads were aligned against a cluster reference using Bowtie2 version 2.2.3 [48]. SNP discovery and genotyping were performed with Freebayes v1.0.2–16 [49]. The resulting dataset after preliminary filtering contained 567,463 SNPs with a minimal read count of 8 and 20% allele difference for heterozygosity.

The resulting SNPs dataset was imported into R 4.3.2 [50] as a vcf file and further filtered in the package SNPfiltR 1.0.1 following the package manual [51, 52]. First, sequences were quality filtered, and genotypes with depth ≥ 5 and genotype quality ≥ 30 were retained. Secondly, genotypes with allele balance 0.25–0.75 for heterozygotes were retained as this value is expected to be 0.5 in real loci. Next, all SNPs with a mean depth above 50 were removed (based on the mean read depth of 18.5 in the dataset). Only biallelic SNPs present in ≥ 75% of individuals with minor allele count ≥ 3 were retained. Subsequently, the dataset was thinned to 1 SNP per 150 bp to reduce the linkage among SNPs. To further reduce the linkage between SNPs, the pruning of SNPs in linkage disequilibrium (LD) was performed with the package dartR 2.9.7: the SNPs were pruned when the same pair of SNPs was in LD in the three predefined populations based on r^2^ threshold of 0.2 [53, 54]. The final dataset contained 4043 SNPs with 14.6% of missing data and was subject to statistical analyses.

The main patterns in the dataset were visualized using Principal Component Analysis (PCA) in the package adegenet 2.1.10 [55]. To explore the presence of significant genetic structure differences among a priori defined populations based on sampled basin we used one-level AMOVA in the package StAMPP 1.6.3 [56]. Pairwise genetic differentiation between the fjords was estimated with the *F*_st_ estimator [57], and pairwise 95% confidence intervals were calculated with 1000 bootstraps in the StAMPP package. Further, genetic structure of the dataset was investigated with Discriminant Analysis of Principal Components (DAPC) with prior *a*-score and cross-validation analyses to find the optimum number of PCs to be retained [58]. Mean allelic richness per population (A_r_), was calculated in the package PopGenReport 3.1 [59, 60]. Observed heterozygosity (H_o_), unbiased expected heterozygosity (uH_e_), and the inbreeding coefficient (F_IS_) were calculated in the package dartR 2.9.7 [53, 54].

## Results

### Thyasirid species composition

**Fig. 2:**
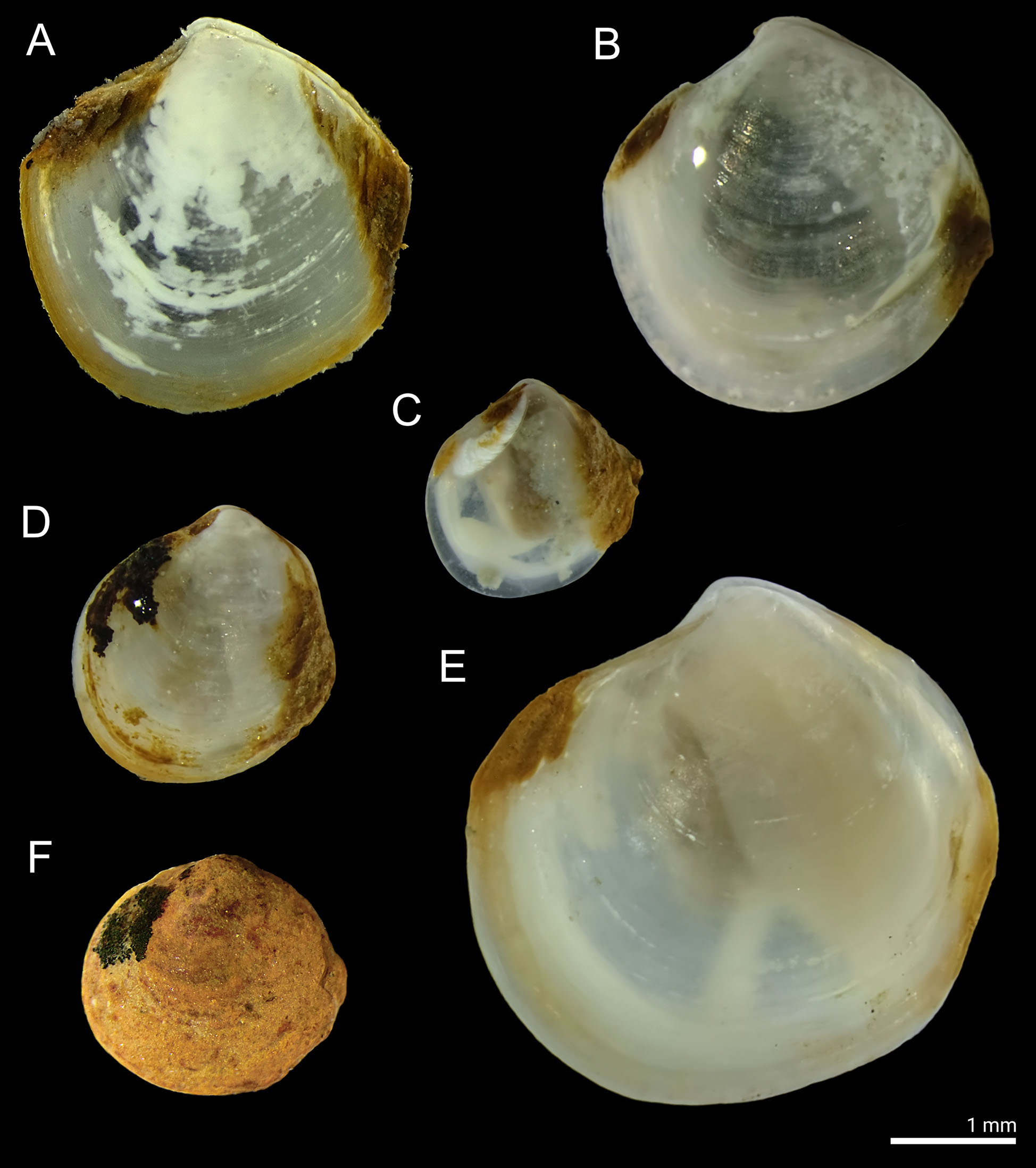
Macrophotographs of thyasirid species studied. (**A**) *Parathyasira equalis*, (**B**) *Parathyasira dunbari*, (**C**) *Genaxinus eumyarius*, (**D**) *Thyasira obsoleta*, (**E**) *Thyasira sarsii*, (**F**) *Mendicula ferruginosa*. Photo credit: Valentin Kokarev

Overall, six thyasirid species were recorded in the three basins (Fig. 2; Table 2). All the morphological identifications were supported by molecular data except for *Parathyasira dunbari*, for which no published sequences currently exist in GenBank. *Parathyasira equalis* was the only species recorded in all three basins. In Saltfjord, it was the only species recorded except for a single individual of *Thyasira obsoleta* in 2015. The most diverse fjord was Sørfolda with 4 species recorded including *Mendicula ferruginosa* and *Genaxinus eumyarius*, which were not found in any other basin sampled. *Parathyasira dunbari* and *Thyasira sarsii* were only recorded in Skjerstadfjord.

Overall, the abundances of *Parathyasira equalis* were higher in Skjerstadfjord compared to Sørfolda and Saltfjord (Table 2). There was an increase in the abundance of *Parathyasira equalis* in 2015 compared to 2013, both in Saltfjord and Skjerstadfjord. There was a noticeable decrease in thyasirid abundance in 2020 compared to 2015 but it must be considered that 2020 samples were processed rapidly and, consequently, some of the bivalves could have been lost, making a direct comparison impossible. In 2020, only 16 specimens of *Parathyasira equalis* were recovered from five grab samples in Sørfolda, while in Skjerstadfjord the number of recovered individuals of this species ranged from 0 to 36 (Supplementary Material 1).

It is worth noting that *Thyasira sarsii* abundances in Skjerstadfjord were low, and there was no noticeable increase in the abundance of this species from 2013 to 2015 as opposed to *Parathyasira equalis* and *Parathyasira dunbari*. During the sampling of 2020, *Thyasira sarsii* individuals were found in the single grab sample, closest to Saltstraumen, where instead of typical silt sediments, a mixture of mud and rocks was recovered along with dead macroalgae and several individuals of large sea urchins, both regular (most probably of the genus *Strongylocentrotus*) and irregular (*Brisaster fragilis*).

**Table 2:** Distribution and abundances (ind./0.1 m^2^) of thyasirid bivalves during the study period. N = number of Van Veen grab (0.1 m^2^) samples. Values expressed as mean ± S.D. note: only presence (+) / absence (-) data are available for 2020

	Saltfjord			Skerstadfjord			Sørfolda	
	2013 (*n* = 16)	2015 (*n* = 6)	2020 (*n* = 6)	2013 (*n* = 22)	2015 (*n* = 6)	2020 (*n* = 6)	2015 (*n* = 22)	2020 (*n* = 5)
*Parathyasira equalis*	0.9 ± 0.9	19.5 ± 4.3	+	18.5 ± 18.3	45.0 ± 13.3	+	14.8 ± 6.6	+
*Parathyasira dunbari*	-	-	-	1.2 ± 1.8	6.7 ± 4.2	+	-	-
*Thyasira obsoleta*	-	0.2 ± 0.4	-	-	-	-	15.7 ± 5.8	+
*Thyasira sarsii*	-	-	-	0.1 ± 0.4	0.8 ± 2.0	+	-	-
*Genaxinus eumyarius*	-	-	-	-	-	-	12.1 ± 8.6	+
*Mendicula ferruginosa*	-	-	-	-	-	-	5.0 ± 3.5	+
Thyasiridae indet.	0.6 ± 1.0	-	-	1.7 ± 2.3	12.8 ± 13.1	-	0.2 ± 0.9	-

### Genetic connectivity of *Parathyasira equalis*

**Fig. 3:**
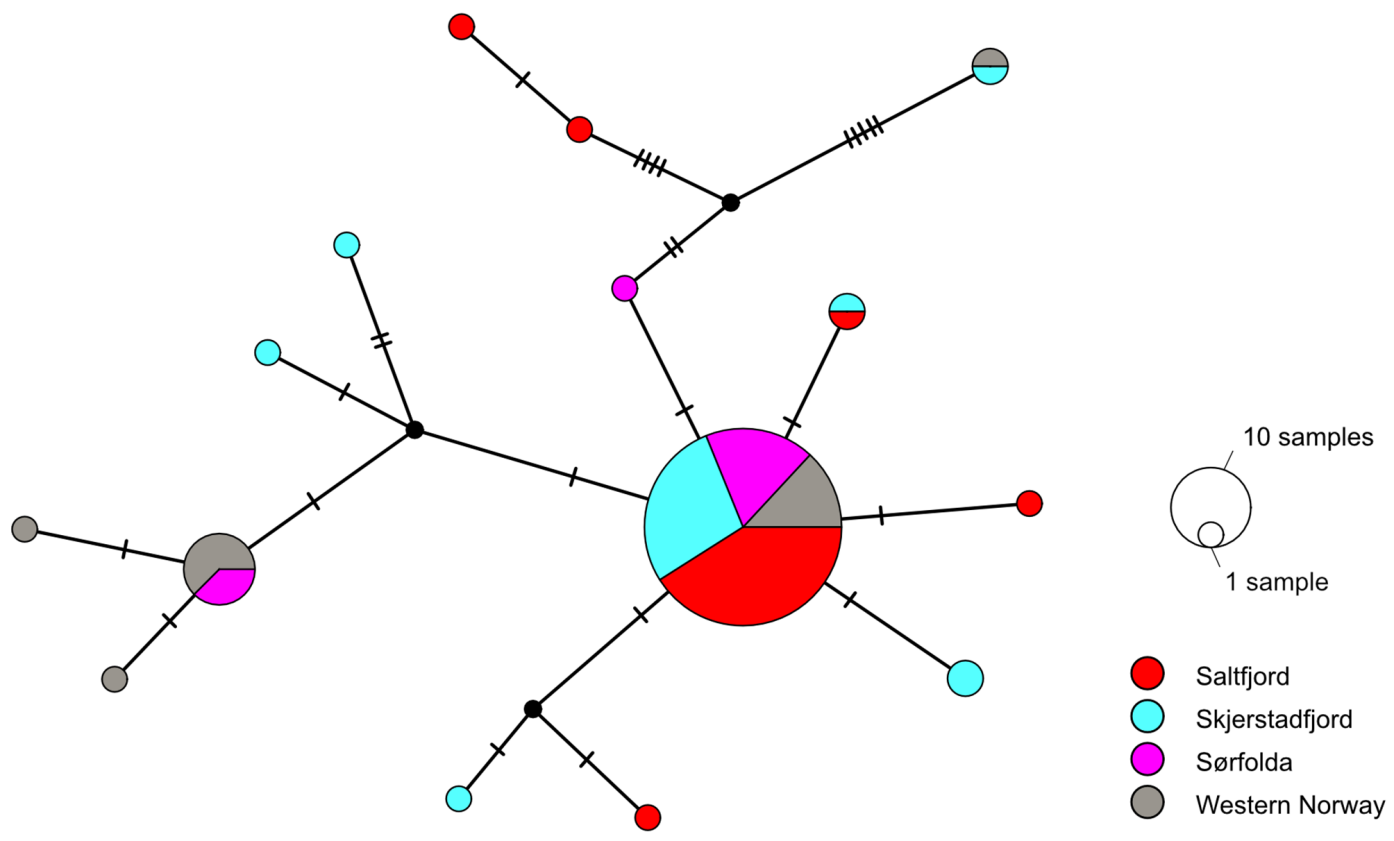
Population genetic structure of *Parathyasira equalis*; TCS haplotype network of the 579 bp COI alignment. Four geographic sites are included: Saltfjord (30 individuals), Skjerstadfjord (24 individuals), Sørfolda (15 individuals), and Western Norway (16 individuals)

Alignment of partial COI sequences revealed 23 total segregating sites with 16 sites being parsimony-informative. Overall, 61 of 85 aligned sequences were identical, while the rest of the haplotypes were represented by a few individuals, and no spatial pattern was evident from the haplotype network (Fig. 3). Consequently, no significant differences were observed between the populations of *Parathyasira equalis* based on the single marker (*Phi*-statistics = 0.00089, *p* = 0.411).

**Fig. 4:**
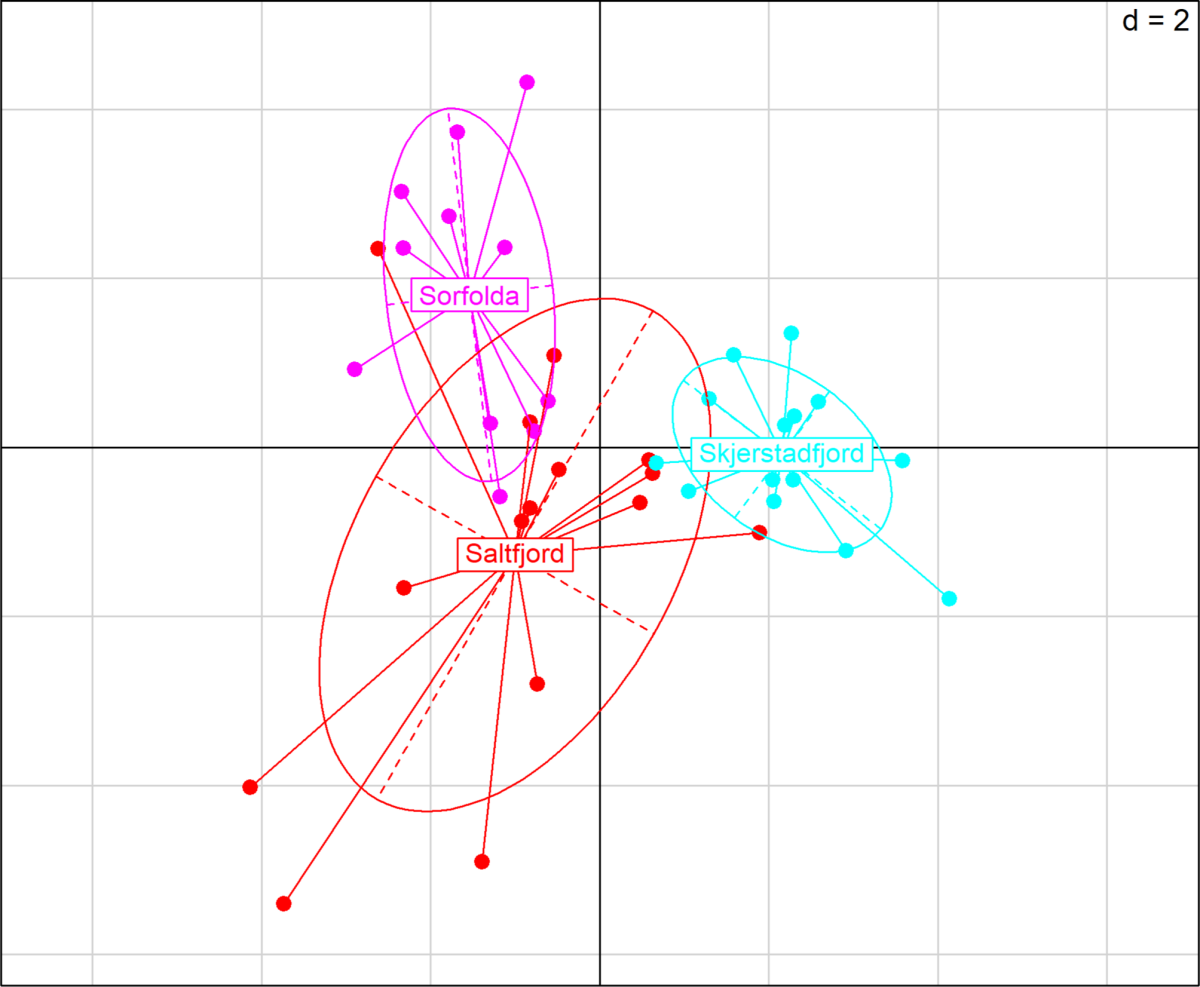
Population genetic structure of *Parathyasira equalis* based on a PCA on 4043 SNPs. The dataset contained 14.6% of missing data. The first and second PCA axes explained 3.2% and 3.0% of the total variation in the dataset, respectively

**Fig. 5:**
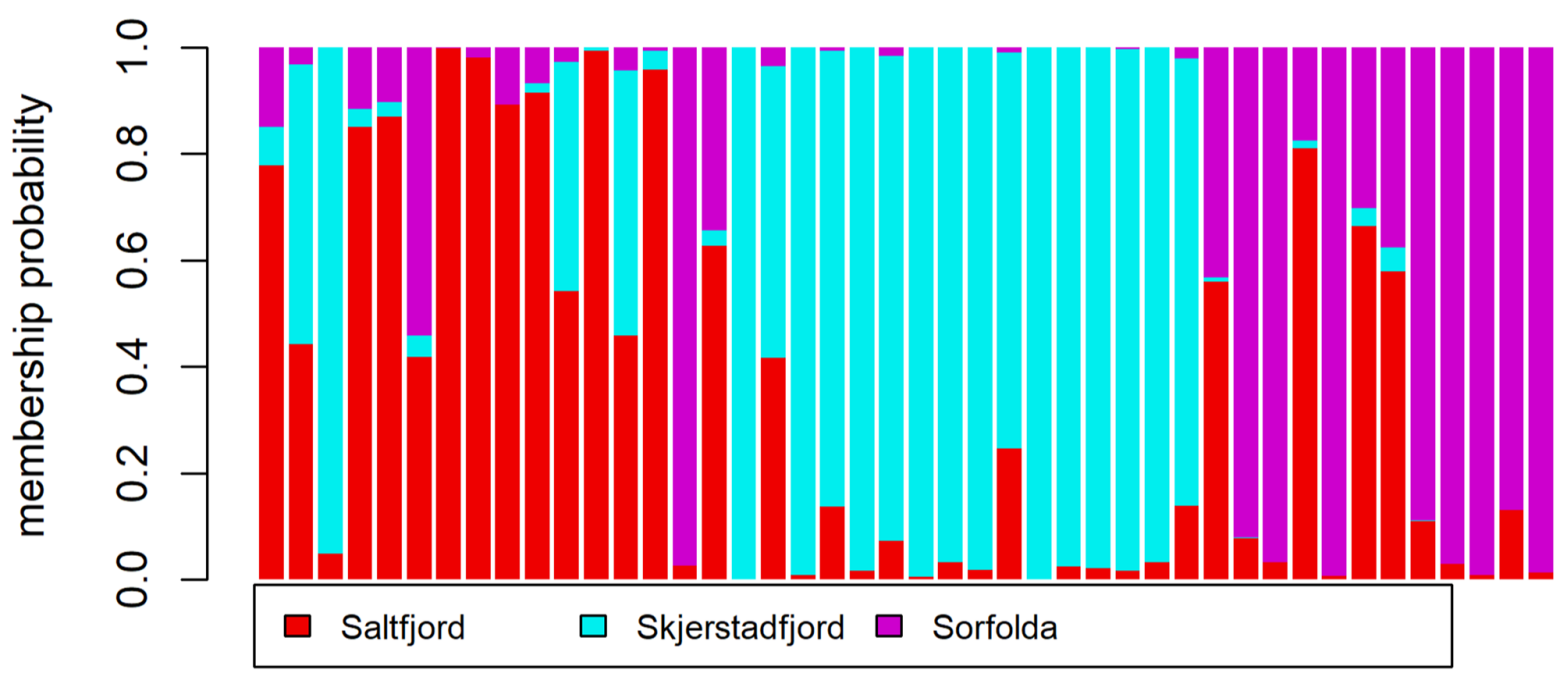
The posterior group assignment probability based on the DAPC. Each column represents a single individual, indicating its estimated membership probability among the sampled populations

In contrast, genetic structure among the populations of the three fjords was significant based on the genome-wide SNPs dataset with 4043 polymorphic sites (AMOVA: *Phi*-statistics = 0.0333, *p* < 0.001). The PCA revealed that Saltfjord had the highest dispersion of samples in the first two principal components, while Skjerstadfjord had the lowest dispersion (Fig. 4). Interestingly, despite their relative remoteness, Saltfjord and Sørfolda were less genetically differentiated relative to Skjerstadfjord based on F_st_ values (Table 3). Higher genetic differentiation of Skjerstadfjord was further supported by the DAPC analysis. For the DAPC analysis, 2 principal components (6.2% of the total variation in the dataset) were retained based on *a*-score and cross-validation analyses, and both resulting discriminant functions were saved. Overall, 16 of 16 (100%) individuals from Skjerstadfjord were successfully reassigned to their original population by DAPC, while this number was 11 of 16 (69%) for Saltfjord and 8 of 12 (67%) for Sørfolda (Fig. 5). Finally, no major differences were observed in terms of genetic diversity in the three populations studied (Ar, Ho, uHe, F_IS_; Table 4).

**Table 3:** Pairwise F_*st*_ values between populations of *Parathyasira equalis* based on 4043 SNPs. The dataset contained 14.6% of missing data. All values were statistically significant (*p* < 0.001)

	Saltfjord	Sørfolda	Skjerstadfjord
Saltford	n/a		
Sørfolda	0.006	n/a	
Skjerstadfjord	0.01	0.014	n/a

**Table 4:** Genetic characteristics of *Parathyasira equalis* populations in the three fjords based on 4043 SNPs. The dataset contained 14.6% of missing data. N = sample size (number of individuals sampled in the population), polyLoci = number of polymorphic loci in the population, monoLoci = number of monomorphic loci in the population, A_r_ = mean allelic richness per population, H_o_ = observed heterozygosity, uH_e_ = unbiased expected heterozygosity, F_IS_ = the inbreeding coefficient

	*N*	polyLoci	monoLoci	A_r_	H_o_	uH_e_	F_IS_
Saltfjord	16	3419	624	1.482	0.117	0.135	0.126
Skjerstadfjord	16	3352	691	1.466	0.115	0.129	0.110
Sørfolda	12	3038	1005	1.458	0.114	0.129	0.113

## Discussion

### Thyasirid species composition

With the exception of *Parathyasira dunbari*, all of the thyasirid species identified in this study were previously recorded along the Norwegian coast and fjords [7, 9, 42]. *Parathyasira dunbari* is widely distributed in the Arctic [61–63] and might not extend its range to lower latitudes in the Atlantic [1, 2]. *Parathyasira dunbari* in Skjerstadfjord occurred at lower densities than *Parathyasira equalis* and might be an example of an “enclaved” population of an arctic species in fjords [64]. Based on [65], *Parathyasira dunbari* (identified by authors as *Thyasira equalis*, but based on the macrophotograph on the Plate VI in the article, the specimen’s identity from the *Bathyarca glacialis* Assemblage-zone can be verified as *Parathyasira dunbari*) was inhabiting fjord basins in Northern Norway already ca. 12,200 year B.P., while boreal species, such as *Thyasira obsoleta* and *Mendicula ferruginosa*, colonised fjords with the inflow of Atlantic waters 10,000 year B.P.

The thyasirid species composition in the three fjords studied remained stable during the whole study period (2013–2020). The only exception was a single specimen of *Thyasira obsoleta* recovered in Saltfjord in 2015. This pattern suggests that populations in fjords are resident, and the advection of individuals from offshore is a rare event, even for the fjords with deep sills. Limited dispersal of thyasirids in the study area corresponds well with an assumption of the short larval pelagic life stage of most thyasirids based on their prodissoconch sizes [1]. Consequently, the increase in thyasirid abundance observed in 2015 in all fjords is most likely related to local recruitment and could be caused by increased food resources. Species in this study are known to have different strategies for obtaining food supply [4, 6, 66, 67]. Species with gills consisting of a single demibranch (*Genaxinus eumyarius* and *Mendicula ferruginosa*) as well as *Thyasira obsoleta* are asymbiotic and therefore need to obtain their nutrition heterotrophically through particulate feeding using their gills, while *Thyasira sarsii* is highly dependent on sulfur-oxidizing symbiotic bacteria in the gills and concentrations of hydrogen sulfide in the sediment. The abundance of chemoautotrophic bacteria in the gills of *Parathyasira equalis* is low, and consequently, a mixed and flexible strategy of heterotrophic/symbiotrophic nutrition can be expected [68]. *Parathyasira dunbari* (determined as *Parathyasira* sp. in Newfoundland fjords, but confirmed to represent the same species based on morphology and unpublished 28S gene sequences) is asymbiotic but might partially rely on the mining of free-living bacteria in the sediments [5, 62, 69]. The 2015 increase in thyasirid abundance was evident for all the species, except *Thyasira sarsii*, and therefore is most probably related to increased organic input from the water column without an associated significant increase in sulfide concentrations in the sediments. Vertical fluxes of organic matter in fjords might show interannual variability depending on complex interactions between physical and biological factors such as wind-driven advection events in and out of fjords, overall primary production as well as zooplankton abundances [70, 71]. Direct observations are necessary to further investigate the relationship between thyasirid abundances and vertical fluxes of organic matter in fjords.

Saltfjord and Sørfolda are quite similar in terms of sediment composition and near-bottom water masses, and possibly water exchange with adjacent coastal waters [8]. The abundance of *Parathyasira equalis* in these two fjords seems also to be similar, at least in 2015. Therefore, it remains unclear why *Genaxinus eumyarius, Thyasira obsoleta*, and *Mendicula ferruginosa* did not establish a population in Saltfjord as there are no apparent environmental or dispersal constraints to this pattern as these species are likely to disperse with the inflow of Atlantic waters similar to *Parathyasira equalis.* A possible explanation might be related to the importance of advection events for fjord ecology which result in increased stochasticity of larval dispersal and vertical fluxes of organic matter in basins. Increased environmental stochasticity might exacerbate the risk of local population extinction or might hamper the establishment of a resident population of these species in Saltfjord [72].

The highest abundances of *Parathyasira equalis* were observed in Skjerstadfjord, which was also the only fjord where *Thyasira sarsii* was observed. This pattern might be related to greater sulfide concentrations in the sediments compared to the other fjords, which would benefit their bacterial symbionts. *Thyasira sarsii* has been suggested as an indicator of organic enrichment and hydrogen sulfide in the sediments [7]. There was no evident increase in the densities of *Thyasira sarsii* in Skjerstadfjord from 2013 to 2015, probably because the concentrations of hydrogen sulfide remained at the same level. In 2020 we observed this species associated with local macroalgal fall at the depth of 362 m. Such hotspots of degrading organic matter might be a source of hydrogen sulfide that supports the population of *Thyasira sarsii* in Skjerstadfjord but overall conditions in the fjord basin do not seem optimal for this species. Another factor that might contribute to the distinctiveness of Skjerstadfjord is its isolation by a shallow sill, and consequently a different pattern of water exchange compared to Saltfjord and Sørfolda, which might influence the vertical fluxes of organic matter to the seafloor through advection.

### Genetic connectivity of *Parathyasira equalis*

The results of the population genetic analyses of *Parathyasira equalis* yielded differed between marker types. Analysis based on the fragment of COI revealed low genetic differentiation along the Norwegian coast. *Parathyasira equalis* is widely distributed in the Atlantic [2], and such a pattern would fit with the large-scale dispersal of individuals with the Atlantic waters into the fjords, which form the bottom water layer in most of the fjords along the Norwegian coast [73].

The use of SNPS generated by GBS might considerably increase the resolution of population genetic studies in bivalves compared to the use of COI sequences [21, 22, 24]. In the present study, GBS revealed small but significant genetic differentiation of populations of *Parathyasira equalis* in the basins with pairwise values of *F*_st_ being higher for Skjerstadfjord. As *F*_st_ is expected to decrease with increased number of migrants per generation [74, 75], it might be assumed that genetic connectivity between Saltfjord and Sørfolda is higher than between Saltfjord and Skjerstadfjord. Given the large geographic distance between Saltfjord and Sørfolda, this high connectivity is most likely not achieved through frequent exchange of individuals. Outflow from the fjords is usually restricted to upper layers [73], which might lead to retention of demersal larvae below the sill depth. Consequently, the populations of *Parathyasira equalis* in Saltfjord and Sørfolda are presumably subpopulations of the same offshore population that extends into these basins with the inflow of Atlantic waters. The higher differentiation of the Skjerstadfjord population is most probably related to the shallow sill, which prevents the inflow of Atlantic waters, and consequently significantly affects the number of migrants into Skjerstadfjord. Overall, low *F*_st_ values (0.006–0.014) and similar genetic diversity estimates indicate that the three populations are closely related, although, it must be noted that the number of migrants needed to sustain gene flow among populations is considerably lower than the number of migrants needed for demographic cohesiveness [37]. Therefore, possible rare advection of individuals sustains genetic connectivity, but this number is too low to affect the basin population demographics. Additional studies, encompassing more fjords and offshore populations of *Parathyasira equalis* are needed to further investigate the relationship between *F*_st_ values and hydrological versus spatial isolation.

## Conclusion

In the present study, we provide evidence that the fjords basins are relatively isolated benthic habitats using thyasirid bivalves as an example. Although our study encompasses only three fjords, two main conclusions can be drawn. First, local reproduction rather than advection of individuals from offshore appears to mainly drive the population dynamics of thyasirid bivalves in fjords. Consequently, the combined effects of environmental perturbations and individual population dynamics due to demographic stochasticity might lead to differences in species composition among otherwise similar habitats [76]. Secondly, significant *F*_st_ values indicate the restricted connectivity among populations of *Parathyasira equalis*, particularly for Skjerstadfjord. These findings are in line with the previous study of these fjords, in which we found a significant differentiation of macrobenthic communities with the Skjerstadfjord community being more distinct compared to Sørfolda and Saltfjord, and the potential role of the shallow sill affecting the community assembly was proposed [8]. Overall, our study highlights the importance of dispersal in structuring macrobenthic communities in accordance with metacommunity theory [11, 12] and highlights that the semi-isolated nature of fjords might be an important driver of local (basin) biodiversity.

### Electronic supplementary material

Below is the link to the electronic supplementary material.

Supplementary Material 1

Supplementary Material 2

Supplementary Material 3

## Data Availability

All the sequences obtained in this study have been uploaded to GenBank and Sequence Read Archive (BioProject: PRJNA1091663). The data on thyasirid abundances in the fjords are available in the electronic supplementary material.
