# Supplementary material for: Thyasirid species composition (Bivalvia: Thyasiridae) and genetic connectivity of Parathyasira equalis (A. E. Verrill & K. J. Bush, 1898) in deep basins of sub-Arctic fjords
Source: BMC Ecol Evol. 2024 Jul 4;24:91. doi: 10.1186/s12862-024-02278-3 (PMC11223403; doi:10.1186/s12862-024-02278-3)
Supplement: Supplementary file 3 — Supplementary Material 3 [file 12862_2024_2278_MOESM3_ESM.docx]

**nGBS library construction on 44 samples of genomic DNA extracted from *Parathyasira equalis* (Bivalvia) using MslI**

**I. Restriction digest:**

30-100 ng of genomic DNA were digested with 2 Units of MslI (NEB) in 1 times Cutsmart buffer in 10µl volume for 30min at 37°C. The restriction enzyme was heat inactivated by incubation at 80°C for 20min.

**II. nGBS library construction:**

**a) Ligation Reaction**

10 µl of each restriction digest were mixed with 1.5 µl of one of 44 inline-barcoded forward MslI Adaptors (pre-hybridized, concentration 5 pM/µl), followed by addition of 20µl Ligation master mix (contains: 15 µl NEB Quick ligation buffer, 0.4 µl NEB Quick Ligase, 5 pM pre-hybridized common reverse MslI Adaptor). Ligation reactions were incubated for 35min at 25°C, followed by heat inactivation for 10 min at 65°C.

**b) Library purification**

all reactions were diluted with 30 µl TE 10/50 (10mM Tris/HCl, 50mM EDTA, pH:8.0) and mixed with 50 µl Agencourt XP beads, incubated for 10 min at RT and placed for 5 min on a magnet to collect the beads. The supernatant was discarded and the beads were washed two times with 200 µl 80% Ethanol. Beads were air dried for 10 min and libraries were eluted in 15 µl Tris Buffer (10 mM Tris/HCl pH:9)

**c) Library amplification**

10 µl of each of the 44 Libraries were separately amplified in 20 µl PCR reactions using MyTaq (Bioline) and standard Illumina TrueSeq amplification primers. Cycle number was limited to 16 Cycles.

**III. Pooling and clean up of ddRAD libraries:**

5 µl from each of the 44 amplified libraries were pooled. PCR primer and small amplicons were removed by Agencourt XP bead purification using 0.8 Volume of beads. The PCR enzyme was removed by an additional purification on Qiagen MinElute Columns. The pooled library was eluted in a final volume of 20µl Tris Buffer (5 mM Tris/HCl pH:9).

**IV. Normalisation**

Normalisation was done using Trimmer Kit (Evrogen). 1 µg pooled GBS library in 12 µl was mixed with 4 µl 4x hybridization buffer, denatured for 3 min at 98°C and incubated for 3 hours at 68°C to allow reassociation of DNA fragments. 20 µl of 2x DSN master buffer was added and the samples were incubated for 10 min at 66°C. One Unit of DSN enzyme (1U/µl) was added and the reaction was incubated for another 40 min. Reaction was terminated by the addition of 20µl DSN Stop Solution, purified on a Qiagen MinElute Column and eluted in 10µl Tris Buffer (5 mM Tris/HCl pH:9).

**V. Reamplification**

The normalized library pool was reamplified in 100µl PCR reactions using MyTaq (Bioline). An i5-Adaptor primer was used to include an i5-Index into the library, allowing parallel sequencing of multiple libraries on the Illumina NextSeq 500/550 sequencer. Cycle number was limited to 14 cycles.

**VI. Size selection**

The GBS library was size selected on a LMP-Agarose gel, removing fragments smaller than 300 bp and those larger than 500 bp.

**VII. Sequencing**

Sequencing was done on an Illumina NextSeq 500/550 v2 (300 cycles).

**Data analysis**

**I. Read pre-processing:**

- Demultiplexing of all library groups using the Illumina bcl2fastq 2.17.1.14 software
  - 1 or 2 mismatches or Ns were allowed in the barcode read when the barcode distances between all libraries on the lane allowed for it
- Demultiplexing of library groups into samples according to their inline barcodes and verification of restriction
  - no mismatches or Ns were allowed in the inline barcodes, but Ns were allowed in the restriction site
- Clipping of sequencing adapter remnants from all reads
  - reads with final length < 20 bases were discarded
- Restriction enzyme site filtering of read 5' ends
  - reads with 5' ends not matching the restriction enzyme site are discarded
- Combination of forward and reverse Restriction enzyme reads using BBMerge 34.48 http://jgi.doe.gov/data-and-tools/bbtools/
- Quality trimming of restriction enzyme Illumina reads (folder 'QualityTrimmed')
  - removal of reads containing Ns
  - trimming of reads at 3'-end to get a minimum average Phred quality score of 20 over a window of ten bases
  - reads with final length < 20 bases were discarded

**II. Clustering, GBS alignment and SNP discovery:**

- Clustering of Restriction enzyme combined reads with CD-HIT-EST v4.6.1, allowing up to 5% difference
  - filtering of the clusters: excluding singletons and clusters created from < 20 reads
- Alignment of quality trimmed reads against cluster reference using Bowtie2 version 2.2.3 (<http://bowtie-bio.sourceforge.net/bowtie2/index.shtml>)
  - one combined alignment for all samples in coordinate-sorted BAM format
- Variant discovery and genotyping of samples with Freebayes v1.0.2-16 (<https://github.com/ekg/freebayes#readme>)
  - The following specific parameters were used: --min-base-quality 10 --min-supporting-allele-qsum 10 --read-mismatch-limit 3 --min-coverage 5 --no-indels --min-alternate-count 4 --exclude-unobserved-genotypes --genotype-qualities --ploidy 2 or 4 --no-mnps --no-complex --mismatch-base-quality-threshold 10
- The SNPs has been filtered for minimum 8 reads to call a SNP and 20% of allele difference for heterozygosity
